# Insulin-Like Peptide Signaling in Mosquitoes: The Road Behind and the Road Ahead

**DOI:** 10.3389/fendo.2019.00166

**Published:** 2019-03-22

**Authors:** Arvind Sharma, Andrew B. Nuss, Monika Gulia-Nuss

**Affiliations:** ^1^Department of Biochemistry and Molecular Biology, University of Nevada, Reno, NV, United States; ^2^Department of Agriculture, Veterinary, and Rangeland Sciences, University of Nevada, Reno, NV, United States

**Keywords:** insulin signaling, insulin-like peptides, mosquitoes, insulin receptor, aedes, anopheles, culex

## Abstract

Insulin signaling is a conserved pathway in all metazoans. This pathway contributed toward primordial metazoans responding to a greater diversity of environmental signals by modulating nutritional storage, reproduction, and longevity. Most of our knowledge of insulin signaling in insects comes from the vinegar fly, *Drosophila melanogaster*, where it has been extensively studied and shown to control several physiological processes. Mosquitoes are the most important vectors of human disease in the world and their control constitutes a significant area of research. Recent studies have shown the importance of insulin signaling in multiple physiological processes such as reproduction, innate immunity, lifespan, and vectorial capacity in mosquitoes. Although insulin-like peptides have been identified and functionally characterized from many mosquito species, a comprehensive review of this pathway in mosquitoes is needed. To fill this gap, our review provides up-to-date knowledge of this subfield.

## Introduction

Insulin-like peptides (ILPs) are broadly conserved among metazoans and are the most studied peptide hormones because of their important regulatory roles in metabolism, growth, and development. All ILPs are 6–8 kDa, share a common structural motif called the insulin fold, and are processed from precursors with similar domain structure (Pre, B, C, A) (1). Among arthropods, insulin signaling is most well-understood in the model insect *Drosophila melanogaster*. The *D. melanogaster* genome has eight ILPs (dILPs), each with specific tissue expression. For instance, some dILPs originate from the brain and ventral nerve cord, while others are expressed in the midgut, fat body, or imaginal discs (2–4).

Mosquitoes have varying numbers of ILPs (Table 1) ranging from five to eight, and, similar to the situation in *D. melanogaster*, expression has been detected in the nervous system, fat body, midgut, ovaries, and other tissues. Each mosquito species has a distinct set of ILPs that are of neural origin, while others are expressed in multiple tissues (5, 7–9).

**Table 1:** Number of insulin-like peptides identified in different mosquito genera and species.

**Mosquito species**	**Number of ILPs**	**References**
*Aedes aegypti*	Eight: Aa ILP1-8	(5)
*Anopheles gambiae*	Five: AgILP1/7, 2, 3/6, 4, 5	(6)
*Anopheles stephensi*	Five: AsILP1-5	(7)
*Culex pipiens*	Three: (more likely)	(8)

Similar to other metazoans, the mosquito insulin receptor (MIR) is a transmembrane receptor tyrosine kinase (RTK) and consists of a dimer of α and β-monomers. The α-subunits define ILP ligand binding specificity, whereas the β-subunits mediate the downstream signal to cellular components. The MIR uses insulin receptor substrate (IRS) as an adaptor molecule to initiate signaling (10). Upon binding of the ligand to its receptor, the β-subunits undergo auto-phosphorylation at specific tyrosine residues. The activated RTK subsequently phosphorylates specific tyrosine residues of the IRS (11). IRS then recruits downstream factors to the receptor-IRS complex. The phosphorylated tyrosine residues of the receptor-IRS complex interact with phosphatidylinositol-3-kinase (PI3K) proteins (12, 13). Recruitment of PI3K results in the formation of the IRS-PI3K complex. Subsequently, PI3K catalyzes synthesis of phosphatidylinositol-3,4,5-trisphosphate (PIP3) from phosphatidylinositol-4,5-bisphosphate (PIP2).

Phosphatase and Tensin homolog (PTEN) is a negative regulator and can reverse this conversion from PIP3 to PIP2 and decrease the level of PIP3 in the cell. The phosphoinositide-dependent protein kinase (PDK) responds to the high PIP3 levels by recruiting Akt (13, 14). Akt is considered the master regulator kinase because the phosphorylation of Akt affects a number of downstream protein substrates including the target of rapamycin (TOR) (15, 16). TOR activation occurs both as a direct downstream event of insulin signaling activation or, independent of Akt, by the availability of amino acids.

TOR and ILP signaling pathways are considered nutritional sensors at the cellular and systemic level, respectively. Akt-mediated phosphorylation of forkhead-related FOXO proteins prevents the FOXO transcription factor from being translocated to the nucleus (17–20). FOXO proteins are indispensable in an organism's response to starvation since they promote conservation of energy or even catabolism (21). There has been some work exploring TOR and FOXO signaling in mosquitoes (22–28), but a detailed review is outside the scope of this article.

## Identification and Structure of ILPs

Prior to the identification of mosquito ILPs and MIR, it was well-known that shortly after blood feeding, neurohormones are released from the brain neurosecretory system that stimulate the ovaries to secrete ecdysteroids, which are necessary for vitellogenesis by the fat body. The silkworm *Bombyx mori* ILP, bombyxin, was demonstrated to stimulate ecdysteroidogenesis in prothoracic glands in silkworm larvae. This led to hypothesis that insulins are involved in regulation of the ecdysteroid pathway in mosquitoes and commercially available porcine and bovine insulin were tested on unfed mosquito ovaries to test this hypothesis. This lead to the discovery of the MIR in *Ae. aegypti* (29) and the discovery of other components of the insulin signaling pathway followed shortly (30). However, ILP identification in *Ae. aegypti* lagged by over half a decade (5). The publication of the *Anopheles gambiae* genome was seminal in the identification of ILPs in mosquitoes. Seven ILP genes corresponding to five unique ILPs (AgILP1-5) and one MIR were identified in the *A. gambiae* genome (9). Two AgILP genes encode identical B and A peptides therefore seven ILP genes produce five peptides. Genes encoding eight unique ILPs were found in the *Ae. aegypti* genome (5). Except for AaILP6, seven other *Ae. aegypti* ILPs had a propeptide structure consistent with that of other invertebrate ILPs. AaILP6 is unique because it has a short C peptide and an extended A peptide, similar to the vertebrate insulin growth factors (IGFs), however, the C peptide of AaILP6 had multiple dibasic proteolytic cleavage sites, in contrast to only one in vertebrate IGFs (5). To date, there is no empirical evidence to confirm whether or not the predicted dibasic sites are actually cleaved/processed in *Ae. aegypti*.

The proximity of AaILP1, 3, and 8 in the genome scaffold suggested that they may form a polycistronic transcription unit controlled by a single promoter. All three of these have independent putative polyadenylation sites and are capped to generate monocistronic mature mRNAs (5). AgILP1/7 and 3/6, the duplicated gene pairs in *A. gambiae*, and AaILP1 and 3 appear to be orthologs. Phylogenetic analysis supported an evolutionary relationship between AaILP1 and AgILP1/7, as well as between AaILP3 and AgILP3/6. A functional relationship between *Anopheles stephensi, Ae. aegypti*, and *Cx. quinquefasciatus* ILP3 was also demonstrated (31). The third member of the *Ae. aegypti* ILP operon, AaILP8, was not related in sequence to any of the other dipteran ILPs (5).

**Figure 1:**
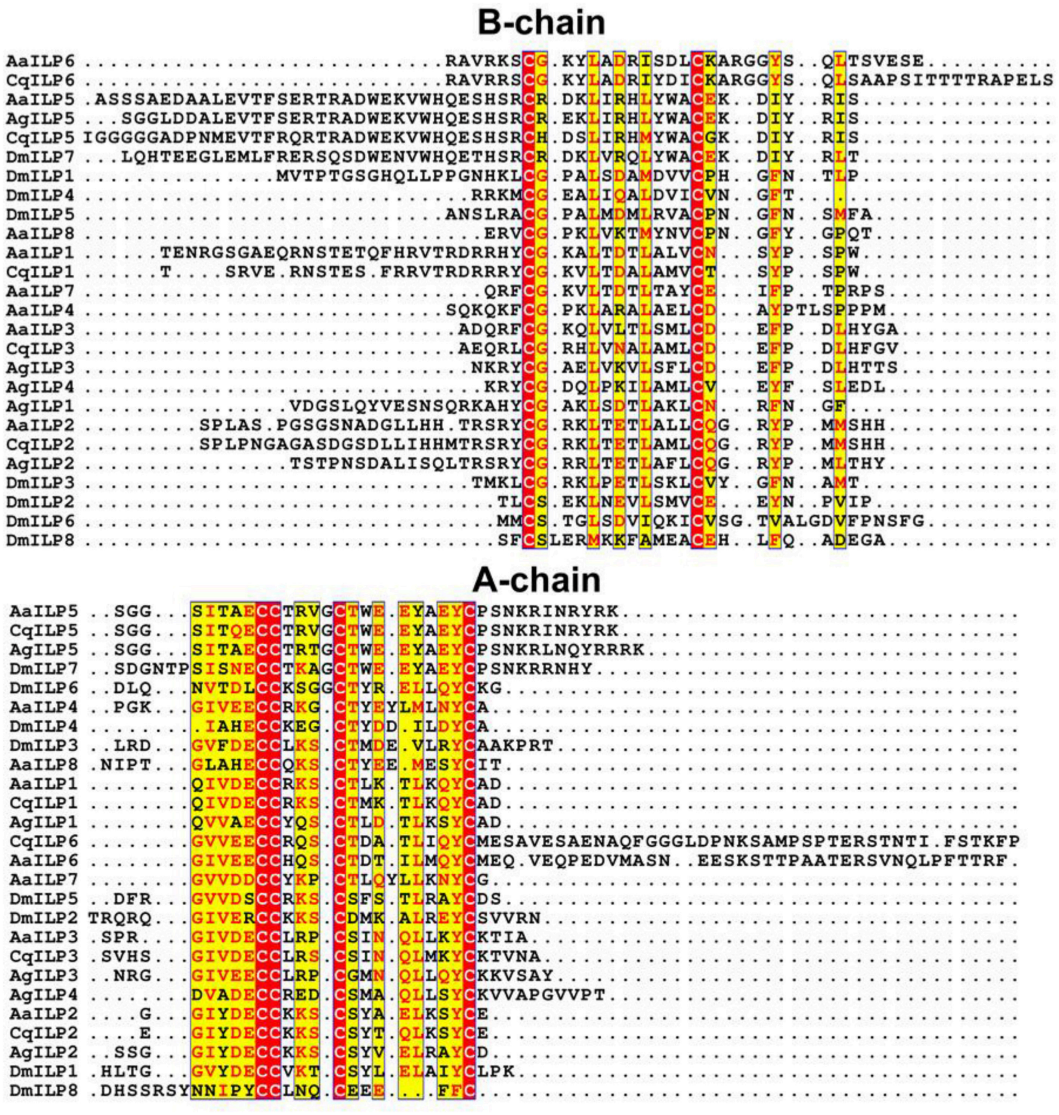
Alignment of mosquito ILP amino acid sequences comparing *Ae. aegypti* (Aa), *C. quinquefasciatus* (Cq), *A. gambiae* (Ag), and *D. melanogaster* (Dm) ILPs by B- or A-chain. Red highlights indicate all identical residues, yellow highlights indicate majority conserved residues (red text indicates conserved residues, black indicates variants). The exceptionally long predicted A-chain of *C. quinquefasciatus* ILP6 is truncated at Pro146 for the sake of space. Alignments were performed in Pôle Rhône-Alpes de Bioinformatique (PRABI) website (32) and ESPript 3.0 (33).

Sequences encoding full-length transcripts of five ILPs from the *A. stephensi* genome (AsILP 1–5) and three from *Culex pipiens* (CpILP1, CpILP2, and CpILP5) were identified (7, 8). ILP4 of *A. stephensi* was highly similar to *A. gambiae* ILP4 but this ILP does not have an apparent ortholog in either *Ae. aegypti* or *C. pipiens*. *Ae. aegypti* ILP5 and *A. gambiae* ILP5 share up to 81% sequence similarity, uncommon for ILPs, and, together with *Cx. pipiens* and *A. stephensi* ILP5, share the unique feature of an additional amino acid between the second and third cysteine residues in the A chain (5, 7). DmILP7, an ortholog of AaILP5, also shares this feature and is well-conserved with the mosquito sequences [(5); Figure 1]. AaILP2 and AgILP2 form another related ILP subgroup (Figure 1).

The putative IGF-like ILP, AaILP6, was closely related to another ILP identified as a gene transcript in *Aedes subalbatus* (5). The remaining two AaILPs, AaILP4 and 7, do not appear to have any dipteran orthologs (Figure 1). This is not surprising considering that both possess an additional amino acid between the third and fourth cysteine residues in the A peptide, a feature not known for other members of the insulin superfamily (5).

**Figure 2:**
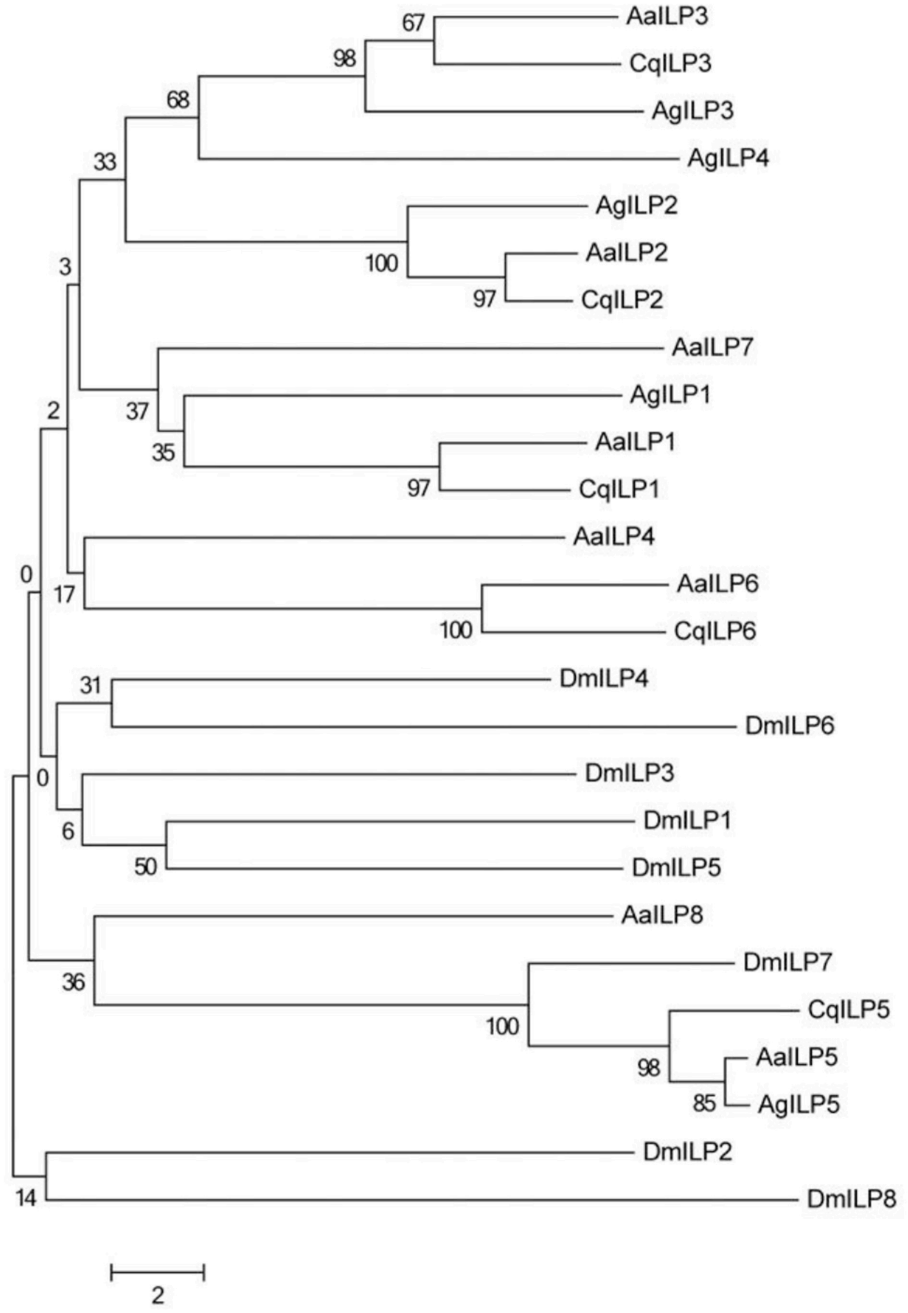
Neighbor-joining tree of mosquito ILP A and B chain amino acid sequences comparing *Ae. aegypti* (Aa), *C. quinquefasciatus* (Cq), *A. gambiae* (Ag), and *D. melanogaster* (Dm). Tree was constructed using MEGA version 6 (34).

A unifying feature of all ILPs is the presence of six conserved cysteine residues that form disulfide bonds between the B and A chains (Figure 1). However, outside of these core residues, amino acid sequence similarity diverges between the different types of ILPs. Some functional forms of ILPs are clearly conserved throughout the mosquito (such as ILP2, ILP3) and even dipteran lineages (such as mosquito ILP5/DILP7), and form distinct groupings when subjected to neighbor-joining analysis (Figure 2), but the evolutionary relatedness of different ILP isoforms to one another has poor branch support and remains unclear. It is likely that the secondary structure imposed by disulfide bonds and as yet undetermined key functional residues are the most critical components for ILP interaction with the MIR, whereas other amino acids may be more important in preserving spacing in the molecule, rather than the identity of their functional group. This limits our ability to predict functions of ILPs in related species based on amino acid sequence alone.

## Functions of Insulin-Like Peptides in Mosquitoes

**Figure 3:**
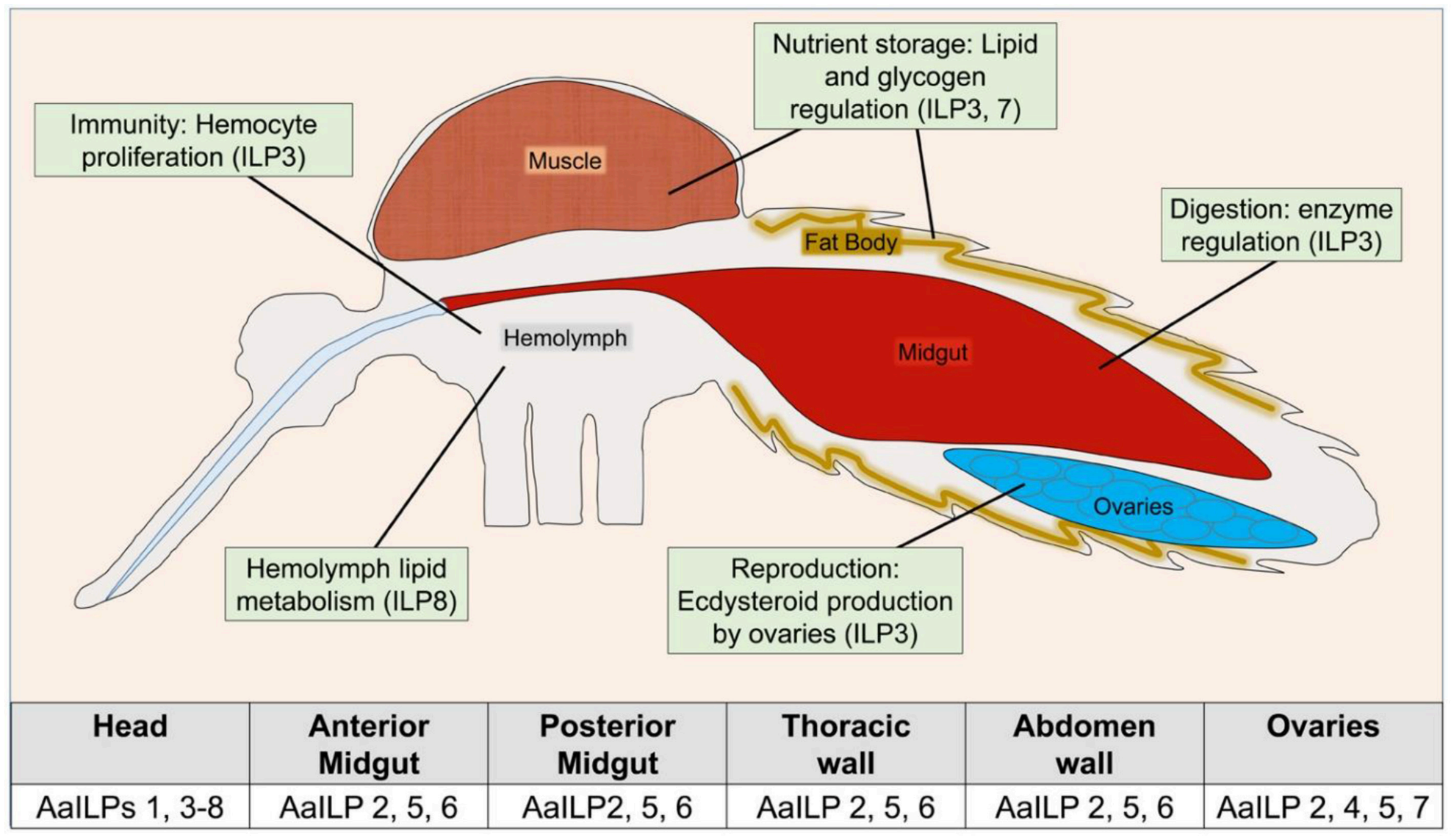
Overview of known ILP functions in *Ae. aegypti*. Table indicates detection of AaILP expression in adult females, as summarized from (5). For a more detailed description of ILP functions in other species, see Table 2.

Function and signaling of ILPs are best characterized for *Ae. aegypti* [for other reviews see (35–37)]. Unlike *D. melanogaster*, genetic manipulations of ILPs to study pluripotency in mosquitoes is still in its infancy. The only native ILP isolated so far from mosquitoes is from *A. stephensi*, AsILP3 (31). AaILP3, AaILP4, and AaILP8 were chemically synthesized (23, 38, 39) and used to deduce their functions. With the availability of new CRISPR-Cas9 based gene editing tools, the functions of two additional *Ae. aegypti* ILPs, AaILP7, and AaILP8, were recently investigated (40) (Table 2, Figure 3).

**Table 2:** Potential functions of mosquito insulin-like peptides.

**Insulin-like peptide (ILP)**	**Potential function**	**References**
***Aedes aegypti***		
AaILP1	Not yet studied	
AaILP2	Not yet studied	
AaILP3	Nutrient metabolism, regulation of digestive enzymes, Ecdysteroid production from ovaries, immune response	(23, 38, 41, 42)
AaILP4	Nutrient metabolism in males	(43)
AaILP5	Not yet studied	
AaILP6	Not yet studied	
AaILP7	Glycogen metabolism post blood meal, nutrient metabolism post blood meal	(40)
AaILP8	Hemolymph lipid metabolism, larval molt	(40, 43)
***Anopheles stephensi***		
AsILP1	Not yet studied	
AsILP2	Not yet studied	
AsILP3	Ecdysteroids production by ovaries	(31)
AsILP4	Ecdysteroids production by ovaries; *Plasmodium falciparum* early infection	(31, 44)
AsILP5	*P. falciparum* oocyst development	(44)
***Anopheles gambiae***		
AgILP1/7	Not yet studied	
AgILP2	Not yet studied	
AgILP3/6	*P. falciparum* infection	(45)
AgILP4	Blood meal nutrients metabolism	(46)
AgILP5	Blood meal nutrients metabolism	(46)
***Culex pipiens***		
CpILP1	Diapause/overwintering	(8)
CpILP2	Not yet studied	
CpILP5	Higher expression but not associated with diapause	(8)

### Nutrient Metabolism

Among mosquitoes, the role of ILPs in nutrient allocation is best studied in *Ae. aegypti*. AaILP1, ILP3, and ILP8 are specifically expressed in the brains of adult females (5). Synthetic AaILP3 (sAaILP3) binds to the MIR with high affinity and has been shown as a critical regulator of egg production (23, 38, 39, 41). sAaILP4 and sAaILP8 did not show any competition with sAaILP3 for binding to the MIR (39). Similarly, sAaILP3 (but not sAaILP4 and sAaILP8) injected into mosquitoes decapitated after a sugar meal dose-dependently increased the levels of stored glycogen and lipids and decreased the levels of trehalose (38) in the whole body of females, suggesting its function is analogous to mammalian insulin in vertebrates.

AaILP3 transcript levels were higher in mosquitoes that emerged from the high carbohydrate larval diet (43), further suggesting a role in nutrient metabolism. AaILP3 also stimulated the midgut to express trypsin-like proteases that digested the blood meal while amino acid sensing through the target of rapamycin (TOR) pathway enhanced AaILP3, ovary ecdysteroidogenic hormone (OEH), ecdysteroids, and vitellogenin synthesis (23, 47).

Some evidence exists that microRNAs regulate AaILP expression. For instance, in the absence of miR277, transcript levels of both AaILP7 and AaILP8 increased in head whereas AaILP1 and AaILP3 transcript levels did not change, suggesting that miR277 targets the first member (AaILP8) of the ILP8-ILP1-ILP3 operon (40). CRISPR-Cas9 depletion of AaILP7 and AaILP8 led to metabolic and reproductive defects. A dramatic lipid increase in the fat body in AaILP7 knockouts and a decrease inAaILP8 knockouts suggests a role of these ILPs in modulating lipid deposition and mobilization (40). Glycogen levels exhibited the opposite trends in these mosquitoes, which suggest that both AaILP7 and AaILP8 are involved in lipid and glycogen balance. In our study, AaILP8 transcript levels were higher in the late fourth instar larvae suggesting a possible role in larval to pupal molt (43), a function similar to *D. melanogaster* ILP8 (2).

In *A. gambiae*, artificial blood meal (albumin and amino acid mixture) rapidly triggered transcription of two ILPs- AgILP3 and AgILP4, in the brain of starved mosquitoes, and the response was higher compared to sucrose fed mosquitoes (46). In *A. stephensi*, expression of ILPs did not change significantly with age or upon ingestion of a sugar or blood meal (7, 44) suggesting differences in mosquito species. ILP functions in nutrient allocation in *Culex* spp. have not been studied yet.

Insulin receptor knockdown by RNA interference (RNAi) in newly eclosed females and subsequent decapitation within 2 h post blood meal resulted in slow blood digestion in *Ae. aegypti* (23). In mosquitoes decapitated post blood meal, sAaILP3 was able to restore trypsin transcripts and enzyme levels, while sAaILP4 and sAaILP8 had no effect on trypsin expression (23). A similar effect of insulin receptor knockdown on midgut trypsin levels was observed in *C. quinquefasciatus* (48). Whether this phenomenon extends to *Anopheles* spp. remains to be explored.

### Reproduction

#### Ecdysteroid and Vitellogenin Production

The first indication of ILP involvement in insect reproduction was the use of bovine insulin to stimulate ecdysteroid production by *in vitro* ovaries isolated from unfed female *Ae. aegypti* (29). Further evidence that this effect was transduced through the insulin signaling complex was provided by using inhibitors or activators of the insulin receptor, PI3K, and Akt, which altered this response (6, 30, 49). Bovine insulin in combination with 20-hydroxyecdysone activated transcription of the yolk protein precursor gene and vitellogenin (Vg) in fat body culture. RNAi-mediated knockdown of the MIR and Akt inhibited insulin-induced Vg gene expression in *in vitro* fat body culture assays (47). sAaILP3 activated ecdysteroid production in unfed ovaries *in vitro* (23, 38, 50). sAaILP4 also stimulated ovaries to produce ecdysteroids *in vitro*, however, five times higher concentrations of sAaILP4 compared to sAaILP3 were required (39).

*Anopheles stephensi* sILP3 and sILP4 were both able to stimulate ovaries to produce ecdysteroids *in vitro* across the genera. Both sAsILPs stimulated ecdysteroid production from unfed ovaries in *A. stephensi, A. gambiae, Ae. aegypti*, and *C. quinquefasciatus* (31) suggesting a conserved role of ILPs in the regulation of ecdysteroid productions in mosquitoes. Insulin receptor knockdown in *C. quinquefasciatus* resulted in low levels ecdysteroids in blood-fed female ovaries (48) further supporting the findings that insulin signaling is required for ecdysteroid production.

#### Yolk Deposition

As the blood meal is digested by the female mosquito and nutrients are mobilized, the developing eggs uptake these nutrients as the yolk. Insulin receptor knockdown resulted in a decrease in the amount of yolk deposited in *Ae. aegypti* ovarioles (23). Injection of sAaILP3 in decapitated, blood-fed females stimulated yolk deposition in ~50% ovarioles (23), whereas sAaILP3 injection in unfed females stimulated yolk deposition in a ~2% ovarioles that were later resorbed and never resulted in egg deposition (41, 51). RNAi knockdown of PTEN, a negative regulator of insulin receptor substrate, in *Ae. aegypti* led to an increase in egg production (52) further supporting the role of insulin signaling in reproduction.

CRISPR-Cas9 mutations of AaILP7 and AaILP8 affect ovarian development, but the phenotypes were different. AaILP7 mutant ovaries and their follicles were similar in size to the wild-type at 24 h post blood meal but were only half the size of those in the control by 72h. These mosquitoes also had elevated lipid stores at 72 h. In contrast, AaILP8 mutant ovaries were small and melanized by 24 h post blood meal (40). In *C. quinquefasciatus* females, insulin receptor knockdown and filarial nematode infection resulted in the complete shutdown of egg maturation and deposition (48).

### Diapause

Diapause is characterized by an arrest in ovarian development and the sequestration of large amounts of lipid reserves. The short day lengths program the temperate mosquitoes such as *C. pipiens* to enter a reproductive diapause. Insulin signaling and FOXO (forkhead transcription factor), a downstream molecule in the insulin signaling pathway, are shown to mediate the diapause response (22). In non-diapausing mosquitoes, RNAi knockdown of the insulin receptor led to primary follicles arrested in a stage comparable to diapause. Juvenile hormone application reversed this diapause-like state. When dsRNA directed against FOXO was injected into mosquitoes programmed for diapause, fat storage was dramatically reduced and the mosquito's lifespan was shortened, suggesting that a shutdown of insulin signaling activates the downstream gene FOXO, leading to the diapause phenotype (22). Transcript levels of CpILP1 and 5 were significantly lower in diapausing females than in their non-diapausing counterparts (8). Knocking down CpILP1 with RNAi in non-diapausing mosquitoes resulted in a cessation of ovarian development similar to diapausing female mosquitoes, whereas CpILP5 did not alter ovarian development (8).

### Lifespan

The first report of the involvement of insulin signaling in lifespan regulation in invertebrates came from work in *Caenorhabditis elegans*. In *C. elegans*, a hypomorphic mutation in the insulin receptor homolog, Daf-2, resulted in a 300% increase in lifespan (53). In *D. melanogaster*, hypomorphic insulin receptor expressing flies showed an 85% increase in lifespan (54). In mosquitoes, overexpression of a myristoylated and active form of *A. stephensi* and *Ae. aegypti* Akt in the fat body of transgenic mosquitoes after blood feeding significantly increased adult survivorship relative to non-transgenic sibling controls (55). Similarly, PTEN overexpression also extended mosquito lifespan (56). Therefore, the effect on lifespan in these experiments with mosquitoes seems to be opposite of that seen in *C. elegans* and *D. melanogaster*, however, the direct effect of insulin receptor knockdown on lifespan has not yet been studied in mosquitoes. The lack of research is partly due to a lack of easily available genetic tools to make hypomorphic insulin receptor expressing mosquito lines. Most work in mosquitoes is done by RNAi, the effect of which lasts only for 7–10 days. In *A. stephensi*, high doses of ingested human insulin with blood meal were shown to reduce lifespan (57–59). In contrast, ingested human IGF1 extended lifespan in this species (60).

## Mosquito Immunity/Mosquito-Pathogen Interactions

Mosquito hemocytes serve as the most important constitutive defense element against pathogens that enter the hemocoel (61, 62) and can produce phagocytic and melanotic immune responses (63, 64), effector molecules (65–69), and enhanced defense associated with immune priming (70). Decapitation of *A. aegypti* mosquitoes after blood feeding inhibited hemocyte proliferation and a single dose of sAaILP3 rescued hemocyte proliferation. Knockdown of the insulin receptor by RNAi inhibited ILP3 rescue activity. This suggests another role of ILPs in hemocyte proliferation, and thus immunity (42).

### Malaria Parasite

The first indication that insulin signaling could play a role in mosquito-pathogen interaction came from a study suggesting human insulin could promote the development of *Plasmodium falciparum* oocysts in the midguts of *A. stephensi* and *A. gambiae*, although the insulin levels used vastly exceeded those in human blood at the physiological levels (71). *P. falciparum* glycosylphosphatidylinositols, a parasite factor that mimics insulin in mammals (72, 73), was later shown to activate insulin receptor, Akt/PKB (Protein kinase B), and the mitogen-activated protein kinase, DSOR 1, in the malaria vector *A. stephensi* (74).

Human IGFs, within a physiological range and higher levels of human insulin, has been shown to induce nitric oxide (NO) synthesis in mosquito cell culture and in the *A. stephensi* midgut (74, 75). Inducible NO synthesis in *A. stephensi* limits malaria parasite development through the formation of inflammatory levels of reactive NO that likely induce parasite apoptosis in the mosquito midgut lumen (57, 74, 76, 77). Both radioactive human insulin and IGF1 persisted intact in the midgut up to 30 h post ingestion and human insulin could activate mosquito insulin receptor by phosphorylation (60).

*P. falciparum*-infected blood meal increased expression of AsILP2, 3, 4, and 5 in the head and midgut of *A. stephensi* (7). Similarly, soluble *P. falciparum* products directly induced AsILP expression in immortalized *A. stephensi* cells *in vitro*. Knockdown of AsILP4 by RNAi induced early expression of immune effector genes within 1–6 h after infection, resulting in significantly reduced parasite abundance prior to invasion of the midgut epithelium. In contrast, knockdown of AsILP3 increased expression of the same genes 24 h after infection. These data suggest that *P. falciparum* parasites alter the expression of mosquito ILPs to blunt the immune response and facilitate parasite development in the mosquito vector (44). *P. berghei* infection significantly increased AsILP3, 4, and 5 expression. Simultaneous knockdown of AsILP3, 4, and 5 by RNAi reduced *P. berghei* development, yet the difference was not statistically significant (78), whereas insulin receptor knockdown in *A. stephensi* significantly reduced *P. berghei* development to oocysts (78).

In transgenic *A. stephensi*, overexpression of Akt in the midgut of heterozygous mosquitoes resulted in 60–99% reduction in the numbers of mosquitoes infected with *P. falciparum*, and parasite infection was completely blocked in homozygous transgenic mosquitoes (79). In addition, a single nucleotide polymorphism (SNP) in the AgILP3 gene (Ins34) was reported in field-collected *A. gambiae* mosquitoes from Mali (45). This synonymous SNP in Ins34 in AgILP3 precursor gene resulted in a change from GGC to GGT at nucleotide position 462. The CC genotype at the Ins34 locus in M form mosquitoes was more common in samples that were not infected with *P. falciparum* suggesting a role of this pathway in malaria parasite infection.

### Filarial Parasites

Insulin receptor knockdown in *C. quinquefasciatus*, the major vector of *Wuchereria bancrofti* in India, completely blocked the development of filarial nematode parasites to the infective third instar larval stage (48). This is the only study on the role of mosquito insulin signaling in nematode development and the data suggest a conserved role of insulin signaling in parasite development within mosquito vectors.

## Conclusions

Insulin-like peptides are pleiotropic peptide hormones, and owing to this, the structural and functional characterization of ILPs has long been a major interest for insect endocrinologists. The functions of insect ILPs, in general, is in a discovery phase compared to the state of knowledge for insulins and related peptides in vertebrates. A primary action of insulin in mammals is to reduce circulating glucose through increased glycogen and triglyceride synthesis. This action is conserved in mosquitoes and has been supported by the work in *Ae. aegypti*. Over the past several years, a succession of studies has suggested a central role of ILPs/insulin signaling in regulating growth, development, reproduction, diapause, aging, and pathogen development in mosquitoes. Yet, only a few studies have used genetic tools to dissect out the functions of individual ILPs. Unraveling the individual functions and functional redundancy of ILPs will provide new understanding of this complex pathway.

## Future Goals

It is clear from the review of literature that there are many unanswered questions regarding the roles of insulin signaling in mosquitoes. Only a few mosquito ILPs have been functionally characterized, mostly because of the challenges in peptide purification and synthesis, efficient transcript knockdown, and potentially overlapping functions. So far only one endogenous mosquito ILP has been purified and characterized, limiting our understanding of the post-translational processing of these molecules, and there is no data on ILPs that may fold similarly to IGF-I, without cleavage of the C-peptide (e.g., AaILP6). In addition, cell-based expression systems have so far been unable to produce biologically active mosquito ILPs, and chemical ILP synthesis has been difficult due to complex formation of disulfide bridges between multiple Cys residues and proper cleavage of the C-peptide required for folding. Both *Ae. aegypti* and *D. melanogaster* research has benefitted from a handful synthetic ILPs, albeit at a high synthesis cost, and availability of more synthetic ILPs would inform ligand-receptor interactions as well as ILP functions. Also, a standard HPLC or GC-MS protocol for measuring ILPs titers would also help improve our knowledge of ILP functions, either as circulating hormones or as a neurotransmitters.

Recent advances in gene editing technologies now allow explorations of ILP functions outside of model organisms. Most notably, CRISPR-Cas9 knock-ins or knock-outs will facilitate acquisition of new knowledge on how ILPs control physiologies such as nutrient storage, lifespan, development, fecundity, host seeking (appetite), regulation of proteases, and immune response. Genetic knockouts allow for persistent, lifelong knockouts or overexpression mutants which could not previously be achieved through RNAi or injection of synthetic peptides. Additionally, the use of an epitope tag such as HA coupled with single guide RNA (sgRNA) in a donor construct along with a fluorescent marker could be a tool for simultaneously tracking ILP expression locations and patterns, even when the hormone itself is knocked out. With the improvement of gene editing in mosquitoes, it will be possible to understand the functions of ILPs in more mosquito species in addition to *Ae. aegypti, An. gambiae*, and *An. stephensi* in order to understand if ILP functions are conserved or change in different mosquito taxa.

An area of research that is clearly open is the role of insulin signaling in mosquito-pathogen interactions. Therefore, research should focus on understanding the diverse functions of ILPs in mosquitoes including mosquito-pathogen interactions. Insulin mimetics that bind to the insulin receptor and block the downstream processes might be a new avenue to explore for mosquito and mosquito-borne disease control.

## Author Contributions

AS, AN, and MG-N wrote the draft manuscript. AN and MG-N wrote the final manuscript.

### Conflict of Interest Statement

The authors declare that the research was conducted in the absence of any commercial or financial relationships that could be construed as a potential conflict of interest.
